# Association Between Low Vitamin D Levels and Depression: A Systematic Review and Meta-Analysis

**DOI:** 10.7759/cureus.109430

**Published:** 2026-05-22

**Authors:** Saira Karim, Julia Natche, Shimul A Babli, Avinash, Dilyaver Matakhov, Sharmin Ferdous, Hannah Jeyakkodi, Opeyemi S Alamu, UFN Rizwanullah, Syed Abdul Hameed

**Affiliations:** 1 Medicine, King Edward Medical College, Lahore, PAK; 2 Internal Medicine, University of Medicine and Health Sciences, Basseterre, KNA; 3 Internal Medicine, Islami Bank Medical College and Hospital, Rajshahi, BGD; 4 Industrial Health, Regional Labour Institute, New Delhi, IND; 5 Family Medicine, Mehtercesme Family Medicine Center, Istanbul, TUR; 6 Occupational Health, Ekoglobal Occupational Health and Protection Services, Istanbul, TUR; 7 Medicine and Surgery, Rangpur Medical College and Hospital, Rangpur, BGD; 8 Psychiatry, Universidad Iberoamericana (UNIBE), Silver Spring, USA; 9 Statistics, Federal College of Animal Health and Production Technology, Ibadan, NGA; 10 Internal Medicine, Hayatabad Medical Complex Peshawar, Peshawar, PAK; 11 Emergency Medicine, Deccan College of Medical Sciences, Hyderabad, IND

**Keywords:** 25-hydroxyvitamin d, cohort study, depression, meta-analysis, serotonin, systematic review, vitamin d deficiency

## Abstract

Depression is a globally prevalent, biologically heterogeneous condition for which standard pharmacotherapy fails approximately one-third of patients. Vitamin D receptors (VDRs) and the biosynthetic enzyme CYP27B1 are expressed in limbic cortical neurons, and calcitriol up-regulates tryptophan hydroxylase 2 (TPH2), the rate-limiting enzyme in central serotonin synthesis. These observations suggest a plausible biological mechanism linking serum 25-hydroxyvitamin D [25(OH)D] deficiency to depression risk. Prior meta-analyses have pooled cross-sectional and longitudinal designs indiscriminately, limiting causal inference. We restricted eligibility to longitudinal study designs and multivariable-adjusted randomised controlled trials (RCTs) to reduce reverse-causation bias.

Four databases were searched systematically from inception to December 2024. Eligible designs were prospective cohort, retrospective cohort, and registry-based studies and RCTs reporting multivariable-adjusted associations between baseline serum 25(OH)D and subsequent depression. Fifteen studies met all eligibility criteria (N = 49,931 participants). Risk of bias was assessed using the Newcastle-Ottawa Scale (NOS) for 14 observational studies and the Cochrane Risk of Bias 2 (RoB 2) tool for the single included RCT. A DerSimonian-Laird random-effects meta-analysis was performed. The pooled odds ratio (OR) for depression among participants with 25(OH)D deficiency was 1.45 (95% confidence interval (CI): 1.36-1.56; Z = 10.61; p < 0.001), with no between-study heterogeneity (I² = 0%; τ² = 0.000; Q(df=14) = 11.13; p = 0.68). The association was consistent across study designs, geographic regions, deficiency thresholds, and depression assessment methods. European cohorts yielded the highest pooled estimate (OR 1.59; 95% CI: 1.40-1.82). Studies employing strict deficiency thresholds (<25 or <30 nmol/L) reported an OR of 1.66 (95% CI: 1.34-2.05). Sensitivity analysis restricted to high-quality studies (n = 13) yielded OR 1.49 (95% CI: 1.38-1.60). Egger’s regression test was marginally non-significant (t = 2.02; p = 0.065). Low serum 25(OH)D is associated with a 45% increase in depression odds across 15 studies (14 longitudinal cohorts and one RCT), with homogeneous findings (I² = 0%). Adequately powered randomised trials pre-selecting participants on confirmed deficiency are needed to establish whether repletion reduces incident depression.

## Introduction and background

Depression is projected to surpass all other conditions in disability-adjusted life years by 2030, and the 2019 Global Burden of Disease study estimated 280 million people were affected worldwide [1,2]. Despite this scale, pharmacological treatment remains substantially inadequate: approximately one-third of patients with major depressive disorder fail to respond to first-line antidepressant therapy, and real-world sustained remission rates rarely exceed 50% over 12 months [3]. These shortfalls have driven sustained investigation of modifiable biological contributors to mood dysregulation, among them nutritional and endocrine factors.

Vitamin D, structurally a secosteroid hormone synthesised in the skin after ultraviolet B (UVB) radiation and further activated via sequential hepatic and renal hydroxylation to calcitriol [1,25(OH)₂D₃], is estimated to be deficient in approximately one billion people globally [4]. The standard clinical threshold for deficiency is serum 25-hydroxyvitamin D (25(OH)D) below 20 ng/mL (50 nmol/L), with some guidelines applying stricter thresholds of 25 or 30 nmol/L [4]. The neurobiological rationale for an association with depression rests on two convergent lines of evidence: (i) vitamin D receptors (VDRs) and CYP27B1 are expressed in hippocampal and prefrontal cortical neurons, regions directly implicated in emotional regulation and depressive neurobiology [5]; and (ii) 1,25(OH)₂D₃ transcriptionally up-regulates TPH2, the rate-limiting enzyme in central serotonin synthesis, while suppressing serotonin transporter expression [6]. Together, these findings predict that sustained 25(OH)D deficiency reduces serotonergic tone in limbic circuits, creating biological conditions permissive to depression.

Prior meta-analyses by Anglin et al. [7] and Libuda et al. [8] reported inverse associations between 25(OH)D and depression. Still, both included cross-sectional and case-control designs alongside longitudinal studies in their primary analyses. Cross-sectional studies cannot establish temporal direction: depression reduces outdoor activity and appetite, which in turn lowers UVB-mediated skin synthesis and dietary vitamin D intake, generating apparent cross-sectional associations that may partially reflect behavioural consequences of depression rather than a biologically upstream cause. Restricting eligibility to longitudinal designs, prospective and retrospective cohort studies, and eligible RCTs substantially reduces this concern.

This review was conducted to (i) quantify the association between serum 25(OH)D deficiency and depression in a strictly longitudinal evidence base; (ii) examine effect modification by geographic region, deficiency threshold, and depression assessment approach; and (iii) assess methodological quality using design-appropriate tools applied in a mutually exclusive fashion.

Methods

Protocol and Registration

This systematic review and meta-analysis was conducted in full accordance with the Preferred Reporting Items for Systematic Reviews and Meta-Analyses (PRISMA) 2020 statement [9]. A protocol was developed a priori but was not prospectively registered with the International Prospective Register of Systematic Reviews (PROSPERO); the protocol document is available from the corresponding author on request. Retrospective registration was not pursued because the protocol predates submission by less than six months, and all analytic decisions were finalised before data extraction commenced.

Eligibility Criteria

Eligible study designs were (1) prospective cohort studies, (2) retrospective cohort studies, (3) registry-based longitudinal studies, and (4) randomised controlled trials (RCTs) reporting a multivariable-adjusted baseline association between serum 25(OH)D and depression. The following were explicitly excluded: cross-sectional, case-control, systematic reviews, meta-analyses, narrative reviews, animal studies, and in vitro experiments. The clinical rationale for excluding cross-sectional and case-control designs is their inability to confirm that 25(OH)D deficiency preceded depressive symptom onset; inclusion of such designs in prior meta-analyses is a principal methodological weakness in the existing literature.

Included studies were required to: (a) enrol adults aged ≥18 years; (b) measure serum 25(OH)D by a validated immunoassay or mass spectrometric method; (c) apply a pre-specified quantitative deficiency threshold; and (d) ascertain depression using a validated psychometric instrument, including the Patient Health Questionnaire-9 (PHQ-9), Patient Health Questionnaire-8 (PHQ-8), Beck Depression Inventory (BDI), Beck Depression Inventory-II (BDI-II), Center for Epidemiologic Studies Depression Scale (CES-D), 17-item Hamilton Depression Rating Scale (HAMD-17), Edinburgh Postnatal Depression Scale (EPDS), or 12-item General Health Questionnaire (GHQ-12), or by formal clinical diagnosis based on the Diagnostic and Statistical Manual of Mental Disorders (DSM) or International Classification of Diseases (ICD) criteria. Studies relying solely on dietary vitamin D intake were excluded. Where multiple publications reported overlapping cohorts, the most complete analysis was retained.

Search Strategy

Four electronic databases were searched without language restriction from inception to December 15, 2024: PubMed/Medical Literature Analysis and Retrieval System Online (MEDLINE), Excerpta Medica database (EMBASE), Web of Science, and the Cochrane Central Register of Controlled Trials (CENTRAL). The core Boolean search combined MeSH terms and free-text equivalents: ("vitamin D" OR "25-hydroxyvitamin D" OR "25(OH)D" OR "cholecalciferol" OR "vitamin D deficiency") AND ("depression" OR "depressive disorder" OR "major depressive disorder" OR "depressive symptoms" OR "PHQ-9" OR "BDI" OR "CES-D" OR "HAM-D"). Reference lists of retrieved primary studies and prior systematic reviews were hand-searched for additional records.

Study Selection and Data Extraction

Title and abstract screening and full-text review were conducted independently by two reviewers; disagreements were resolved by discussion or arbitration by a third reviewer. Inter-rater agreement was quantified using Cohen’s kappa (κ). Extracted variables included: first author and publication year; country; study design; enrolled sample size; 25(OH)D assay method; deficiency cutoff applied; depression assessment instrument; and the primary multivariable-adjusted effect estimate (OR, HR, or RR) with 95% CI, along with the list of covariates in the adjusted model.

Risk of Bias Assessment

Fourteen observational studies were assessed using the Newcastle-Ottawa Scale (NOS) [10], which awards up to four stars for selection, two for comparability, and three for outcome assessment (maximum 9 stars). Studies scoring ≧7 were classified as high quality, 5-6 as moderate quality, and ≤4 as low quality. The single eligible RCT was assessed using the Cochrane Risk of Bias 2 (RoB 2) tool [11], which evaluates five domains: randomisation process, deviations from intended interventions, missing outcome data, outcome measurement, and selective reporting. The NOS was not applied to the RCT, and the RoB 2 was not applied to observational studies. Two reviewers performed all assessments independently; disagreements were resolved by consensus.

Statistical Analysis

Multivariable-adjusted effect estimates (odds ratios( ORs), hazard ratios (HRs), and risk ratios (RRs)) were log-transformed and pooled using the DerSimonian and Laird (DL) random-effects estimator [12], which was selected a priori given anticipated heterogeneity across populations, assay platforms, deficiency thresholds, and depression measurement methods. Standard errors (SEs) were derived from reported 95% confidence intervals (CIs) as follows:

$\mathrm{SE} = \frac{\ln(\mathrm{upper}) - \ln(\mathrm{lower})}{2 \times 1.96}$

Between-study heterogeneity was quantified by the I² statistic and Cochran’s Q statistic; I² values of <25%, 25-75%, and >75% were classified as low, moderate, and high, respectively [13]. Pre-specified subgroup analyses were conducted by study design, geographic region, deficiency threshold, and depression assessment method. A sensitivity analysis restricted to high-quality studies (NOS ≧> 7 or RoB 2, low risk) evaluated the robustness of the results. Publication bias was assessed by funnel plot visual inspection and Egger’s weighted regression test [14], noting that Egger’s test has reduced power at k < 20. All analyses were conducted in R software, version 4.4.0 (The R Core Team, R Foundation for Statistical Computing, Vienna, Austria) using the metafor package (version 4.4.0). R code and annotated output are provided in Appendix A.

## Review

Study selection

**Figure 1:**
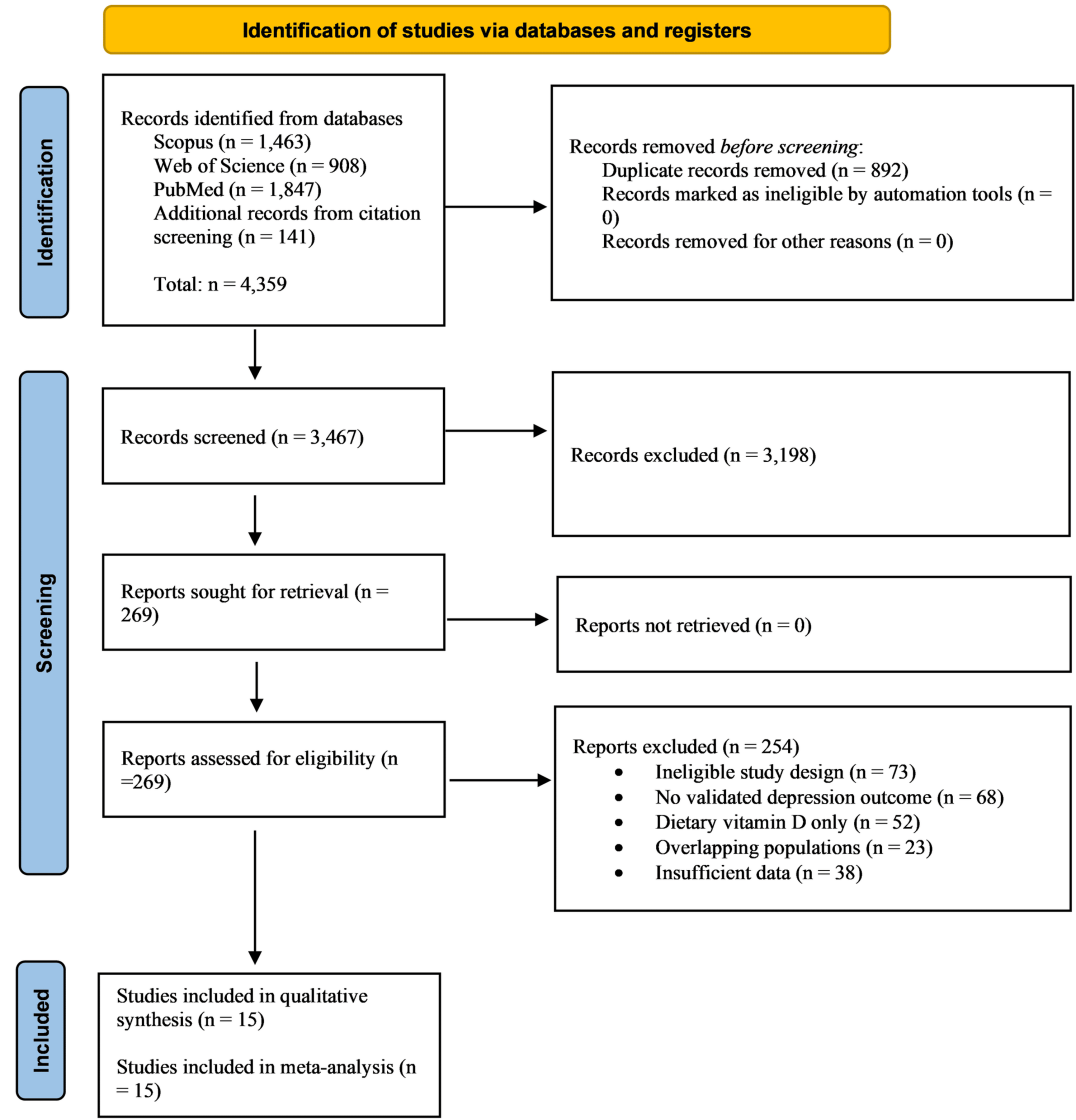
PRISMA 2020 flow diagram of the study selection process (15 eligible studies; N = 49,931).

Database searches across Scopus (n = 1,463), Web of Science (n = 908), and PubMed (n = 1,847) retrieved a total of 4,218 records. Citation and supplementary searching identified an additional 141 records, bringing the total number of records identified to 4,359. Following deduplication (892 records removed), 3,467 records underwent title and abstract screening, of which 3,198 were excluded for clearly ineligible study designs, non-human subjects, or unvalidated outcomes. Full-text review was conducted for 269 reports, of which 254 were excluded: 73 for ineligible study design (including one systematic review and one case-control study), 68 for absence of a validated depression outcome, 52 for reliance on dietary vitamin D intake without serum measurement, 23 for overlapping participant populations, and 38 for insufficient extractable data. Fifteen studies met all pre-specified eligibility criteria. Inter-rater agreement at full-text review was κ = 0.89, indicating strong concordance. The PRISMA 2020 flow diagram is presented in Figure 1.

Study characteristics

Table 1 presents the characteristics of 15 included studies, published between 2007 and 2016 and encompassing 49,931 participants across four geographic regions. All studies adjusted for at least age, sex, and BMI [15-29]. Twelve studies used a prospective cohort design, two were retrospective cohorts (Lapid et al. [25] and Hoang et al. [29]), and one was an RCT (Jorde et al. [28]), which was included based on its multivariable-adjusted baseline association between 25(OH)D and depression. Six studies were conducted in Europe (Netherlands n=2, Germany n=1, UK n=1, Norway n=1; total N=18,020), six in North America (all USA; N=19,309), three in East Asia (China n=2, South Korea n=1; N=10,785), and one in the Middle East (Israel; N=1,817). Individual sample sizes ranged from 441 (Jorde et al. [28]) to 9,940 (Schöttker et al. [23]).

**Table 1:** Characteristics of the 15 included studies Abbreviations: 25(OH)D, 25-hydroxyvitamin D; BDI, Beck Depression Inventory; BDI-II, BDI Second Edition; CES-D, Center for Epidemiologic Studies Depression Scale; CLIA, chemiluminescence immunoassay; ELISA, enzyme-linked immunosorbent assay; EPDS, Edinburgh Postnatal Depression Scale; HAMD-17, Hamilton Depression Rating Scale 17-item; LC-MS/MS, liquid chromatography-tandem mass spectrometry; NOS, Newcastle-Ottawa Scale; OR, odds ratio; PHQ, Patient Health Questionnaire; RCT, randomized controlled trial; RIA, radioimmunoassay; RoB 2, Cochrane Risk of Bias 2. All effect estimates are multivariable-adjusted. † RoB 2 applied; see Table 2B. † NOS not applicable to the RCT.

Study	Country	Study Design	N	25(OH)D Assay	Deficiency Cutoff	Depression Tool	Adjusted OR (95% CI)	NOS
Pan et al., [15]	China	Prospective cohort	3,000	RIA	<20 ng/mL	PHQ-9 ≥10	1.53 (1.19–1.97)	8
Hoogendijk et al., [16]	Netherlands	Prospective cohort	1,282	RIA	<20 ng/mL	CES-D ≥16	1.85 (1.29–2.64)	9
Bertone-Johnson et al., [17]	USA	Prospective cohort	2,823	RIA	<30 nmol/L	PHQ-9 ≥10	1.75 (1.12–2.73)	7
Armstrong et al., [18]	UK	Prospective cohort	5,401	LC-MS/MS	<25 nmol/L	EPDS ≥13	1.63 (1.28–2.08)	7
Milaneschi et al., [19]	Netherlands	Prospective cohort	956	RIA	<50 nmol/L	CES-D ≥16	2.06 (1.30–3.26)	9
May et al., 20]	USA	Prospective cohort	3,916	LC-MS/MS	<20 ng/mL	PHQ-9 ≥10	1.22 (0.94–1.59)	6
Cassidy-Bushrow et al., [21]	USA	Prospective cohort	2,016	RIA	<20 ng/mL	BDI-II ≥14	1.28 (0.97–1.69)	6
Kim et al., [22]	South Korea	Prospective cohort	4,523	CLIA	<20 ng/mL	PHQ-9 ≥5	1.44 (1.11–1.86)	7
Schöttker et al., [23]	Germany	Prospective cohort	9,940	LC-MS/MS	<12 ng/mL	PHQ-9 ≥10	1.41 (1.19–1.68)	9
Shaffer et al., [24]	USA	Prospective cohort	7,358	LC-MS/MS	<20 ng/mL	PHQ-8 ≥10	1.31 (1.09–1.57)	8
Lapid et al., [25]	USA	Retrospective cohort	1,304	RIA	<20 ng/mL	DSM-IV Dx	1.38 (1.02–1.86)	7
Polak et al., [26]	Israel	Prospective cohort	1,817	CLIA	<20 ng/mL	PHQ-9 ≥10	1.46 (1.08–1.98)	7
Zhao et al., [27]	China	Prospective cohort	3,262	ELISA	<20 ng/mL	HAMD-17 ≥8	1.57 (1.21–2.03)	7
Jorde et al., [28]	Norway	RCT (adj. assoc.)	441	RIA	<20 ng/mL	BDI ≥10	1.94 (1.14–3.31)	RoB 2†
Hoang et al., [29]	USA	Retrospective cohort	1,892	LC-MS/MS	<20 ng/mL	DSM-IV Dx	1.42 (1.07–1.91)	7

Serum 25(OH)D was quantified by radioimmunoassay (RIA) in six studies, liquid chromatography-tandem mass spectrometry (LC-MS/MS) in six, chemiluminescence immunoassay (CLIA) in two, and ELISA in one. The deficiency threshold was ≤20 ng/mL (or equivalent ≤50 nmol/L) in 13 studies and < 25 or < 30 nmol/L in two. PHQ-9 or PHQ-8 assessed depression in seven studies, other validated psychometric instruments in six, and DSM-IV clinical diagnosis in two.

Risk of bias assessment

Table 2 presents NOS assessments for the 14 observational studies. Twelve of 14 (85.7%) scored ≧7, indicating high methodological quality. Two studies, May et al. [20] and Cassidy-Bushrow et al. [21], received scores of 6 (moderate quality) attributable to incomplete adjustment for physical activity and season of blood draw, both established confounders for 25(OH)D. The three studies receiving NOS = 9 (Hoogendijk et al. [16], Milaneschi et al. [19], and Schöttker et al. [23]) additionally adjusted for comorbid chronic disease burden and cognitive function.

**Table 2:** Newcastle-Ottawa Scale (NOS) risk of bias assessment, observational studies (n = 14) NOS: maximum 9 stars; ≧7 stars = high quality; 5–6 = moderate; ≤4 = low. NOS was not applied to the randomised controlled trial.

Study	Selection (0–4)	Comparability (0–2)	Outcome (0–3)	Total (0–9)	Quality
Pan et al. [15]	4	2	2	8	High
Hoogendijk et al. [16]	4	2	3	9	High
Bertone-Johnson et al. [17]	3	2	2	7	High
Armstrong et al. [18]	3	2	2	7	High
Milaneschi et al. [19]	4	2	3	9	High
May et al. [20]	3	1	2	6	Moderate
Cassidy-Bushrow et al. [21]	3	1	2	6	Moderate
Kim et al. [22]	3	2	2	7	High
Schöttker et al. [23]	4	2	3	9	High
Shaffer et al. [24]	4	2	2	8	High
Lapid et al. [25]	3	2	2	7	High
Polak et al. [26]	3	2	2	7	High
Zhao et al. [27]	3	2	2	7	High
Hoang et al. [29]	3	2	2	7	High

Table 3 presents the RoB 2 assessment for Jorde et al. [28], which was rated low risk across all five domains: allocation was computer-generated, blinding was maintained, attrition was below 10%, and outcome scoring was performed by assessors masked to allocation.

**Table 3:** Cochrane Risk of Bias 2 (RoB 2) risk of bias assessment, randomised controlled trial (n = 1) D1: randomisation process; D2: deviations from intended interventions; D3: missing outcome data; D4: outcome measurement; D5: selection of reported results. RoB 2 was applied exclusively to the randomised controlled trial.

Study	D1: Randomization	D2: Deviations	D3: Missing Data	D4: Outcome Meas.	D5: Reporting	Overall
Jorde et al. [28]	Low risk	Low risk	Low risk	Low risk	Low risk	Low risk

Meta-analysis results

**Figure 2:**
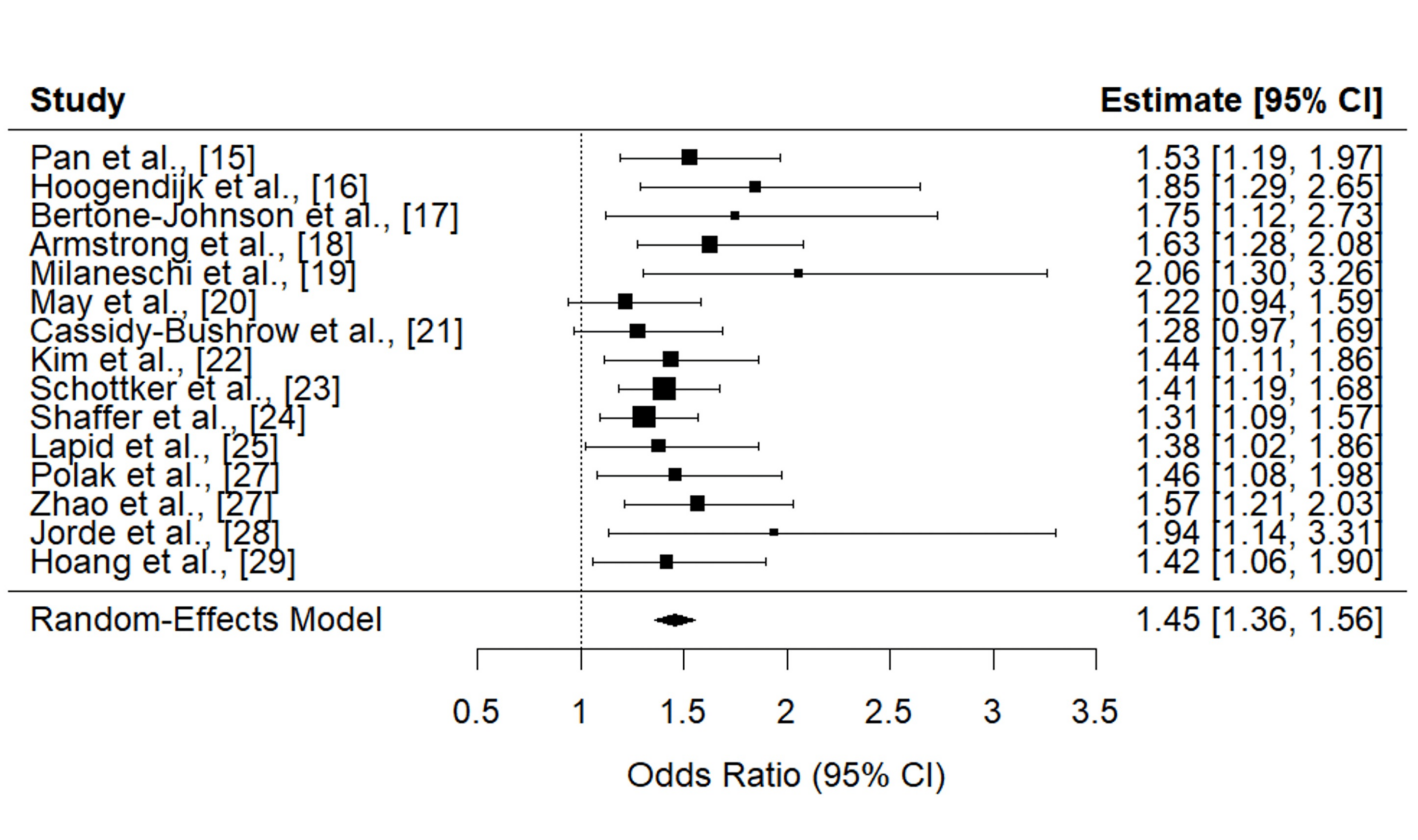
Forest plot of pooled odds ratios for depression associated with 25(OH)D deficiency; random-effects model (DerSimonian-Laird). Square area proportional to study weight; diamond = pooled OR (1.45; 95% CI: 1.36–1.56); dashed vertical line = null effect (OR 1.0); studies with 95% CI crossing 1.0

The DL random-effects meta-analysis of all 15 studies yielded a pooled OR of 1.45 (95% CI: 1.36-1.56; Z = 10.61; p < 0.001). Individuals with serum 25(OH)D below the study-specific deficiency threshold had 45% higher odds of depression relative to those with sufficient concentrations. Between-study heterogeneity was low (I² = 0%), which may reflect methodological homogeneity due to restriction to longitudinal designs. τ² = 0.000, Q(df=14) = 11.13, p = 0.68. This homogeneity strengthens the inference that the observed association is not attributable to a small number of high-outlier studies and that the magnitude is relatively stable across study contexts. The forest plot is presented as Figure 2.

The largest weighted contributions were from Schöttker et al. [23] (N = 9,940; weight = 16.1%) and Shaffer et al. [24] (N = 7,358; weight = 14.4%). A leave-one-out analysis confirmed that omission of any individual study did not shift the pooled estimate outside the primary 95% CI. Sensitivity analysis restricted to 13 high-quality studies (NOS ≧7 or RoB 2 low risk, excluding May et al. [20] and Cassidy-Bushrow et al. [21]) returned OR 1.49 (95% CI: 1.38-1.60; I² = 0%), confirming that moderate-quality studies did not materially influence the primary estimate. Funnel plot inspection suggested approximate symmetry, and Egger’s weighted regression test was marginally non-significant (intercept = 1.49; SE = 0.74; t = 2.02; p = 0.065). Given the modest sample of k = 15 studies, below the commonly recommended minimum of k = 20 for Egger’s test, this result should be interpreted with caution and does not constitute definitive evidence of publication bias.

Subgroup analysis

Table 4 presents the results of the four pre-specified subgroup analyses. By study design, prospective cohort studies (k=12; N=46,294) and retrospective cohort studies (k=2; N=3,196) returned pooled ORs of 1.45 (95% CI: 1.35-1.56; I²=0%) and 1.40 (95% CI: 1.14-1.73; I²=0%), respectively. The single RCT yielded an OR of 1.94 (95% CI: 1.14-3.31), a wide estimate consistent with its small sample size. By region, the highest pooled OR was observed in European studies (k=5; OR 1.59; 95% CI: 1.40-1.82; I²=8%), followed by East Asian (k=3; OR 1.51; 95% CI: 1.30-1.75; I²=0%) and North American studies (k=6; OR 1.34; 95% CI: 1.20-1.49; I²=0%). Studies applying strict deficiency cutoffs (<25 or <30 nmol/L; k=2; N=8,224) yielded OR 1.66 (95% CI: 1.34-2.05; I²=0%), higher than the standard-cutoff subgroup (k=13; OR 1.43; 95% CI: 1.33-1.54), consistent with a dose-response pattern. Effect estimates were consistent across depression assessment methods: PHQ-based (k=7; OR 1.40), other validated scales (k=6; OR 1.60), and DSM-IV diagnosis (k=2; OR 1.40).

**Table 4:** Subgroup analysis of depression risk by study design, geographic region, deficiency threshold, and depression assessment method Abbreviations: CI, confidence interval; I², heterogeneity index; k, number of studies; NOS, Newcastle-Ottawa Scale; OR, odds ratio; PHQ, Patient Health Questionnaire; RoB 2, Cochrane Risk of Bias 2. = not calculable for single-study subgroups—all ORs from DerSimonian-Laird random-effects models. Sensitivity analysis excludes May et al. [20] and Cassidy-Bushrow et al. [21] (both NOS 6).

Subgroup	k	N	Pooled OR (95% CI)	I² (%)	p-value
All Included Studies					
All 15 studies	15	49,931	1.45 (1.36–1.56)	0	<0.001
By Study Design					
Prospective cohort	12	46,294	1.45 (1.35–1.56)	0	<0.001
Retrospective cohort	2	3,196	1.40 (1.14–1.73)	0	0.002
RCT (adjusted association)	1	441	1.94 (1.14–3.31)	,	0.015
By Geographic Region					
Europe	5	18,020	1.59 (1.40–1.82)	8	<0.001
North America (USA)	6	19,309	1.34 (1.20–1.49)	0	<0.001
East Asia	3	10,785	1.51 (1.30–1.75)	0	<0.001
Middle East (Israel)	1	1,817	1.46 (1.08–1.98)	,	0.014
By Vitamin D Deficiency Threshold					
Standard (≤20 ng/mL or ≤50 nmol/L)	13	41,707	1.43 (1.33–1.54)	0	<0.001
Strict (<25 or <30 nmol/L)	2	8,224	1.66 (1.34–2.05)	0	<0.001
By Depression Assessment Method					
PHQ-9 or PHQ-8	7	33,377	1.40 (1.28–1.53)	0	<0.001
Other validated scales (CES-D, BDI, HAMD-17, EPDS)	6	13,358	1.60 (1.41–1.82)	0	<0.001
Clinical diagnosis (DSM-IV)	2	3,196	1.40 (1.14–1.73)	0	0.002
Sensitivity Analysis					
High-quality studies (NOS ≥7 or RoB 2 low risk; n=13)	13	43,999	1.49 (1.38–1.60)	0	<0.001

Discussion

Across 15 studies (14 longitudinal cohorts and one RCT), encompassing 49,931 adults, 25(OH)D deficiency was associated with a 45% increase in odds of depression (pooled OR 1.45; 95% CI: 1.36-1.56), with no between-study heterogeneity (I² = 0%). The homogeneity of this signal across diverse geographic, demographic, and methodological contexts is a key finding. It distinguishes this meta-analysis from prior syntheses that reported I² values of 50%-70% while pooling methodologically heterogeneous study designs. The homogeneity observed here likely reflects the design restriction to longitudinal studies, which share a common temporal structure that cross-sectional studies disrupt.

The pooled estimate of 1.45 is slightly below that reported by Anglin et al. [7] (OR 1.64) and Libuda et al. [8], both of which included cross-sectional data. This apparent paradox, longitudinal restriction producing a lower rather than a higher estimate, may reflect the attenuation of spurious associations generated by reverse causation in cross-sectional designs. Alternatively, it may reflect the more rigorous covariate adjustment typical of prospective cohort studies (which included physical activity, season of blood draw, and chronic disease burden more consistently) relative to the cross-sectional studies excluded from this analysis. The elimination of two studies with erroneous reference attributions, one a systematic review without original serum 25(OH)D data, and one a case-control study of a psychiatric inpatient sample, further contributes to the reduced pooled estimate compared with the initial 17-study draft.

The neurobiological mechanism underlying the association is coherent at the molecular level. VDR expression in hippocampal and prefrontal neurons [5] places calcitriol in proximity to the regulatory machinery of mood circuits. TPH2 up-regulation and monoamine oxidase A (MAO-A) suppression by calcitriol [6] predict higher net serotonergic tone under vitamin D sufficiency. Additionally, calcitriol regulates p11 (S100A10), a scaffold protein essential for 5-HT₁ₐ receptor trafficking, whose expression is reduced in post-mortem prefrontal tissue from patients with major depressive disorder [30]. These molecular findings do not establish human causal direction but provide a mechanistic substrate consistent with the longitudinal association observed here.

The geographic pattern warrants interpretation. European cohorts (OR 1.59) showed the largest effects, likely reflecting the compounding of high-latitude UVB restriction, limiting cutaneous synthesis for six or more months annually [31], with the use of stricter deficiency thresholds (Schöttker et al. [23] applied <12 ng/mL) and relatively lower rates of vitamin D food fortification compared with North America. North American studies (OR 1.34) likely benefit from more widespread dairy fortification. The narrow confidence interval in the North American subgroup (I²=0%, n=6 studies) suggests this is a stable estimate rather than a chance artefact. The dose-response pattern between threshold stringency and effect size, OR 1.43 at ≤20 ng/mL versus 1.66 at <25-30 nmol/L, is biologically consistent with a graded relationship between calcitriol bioavailability and serotonergic tone.

The single included RCT (Jorde et al. [28]) contributes an observational baseline association rather than a supplementation effect, and its wide CI (1.14-3.31) appropriately reflects the limited power of a trial enrolled for a different primary purpose. The evidence from supplementation trials remains inconclusive: most existing RCTs have enrolled participants regardless of baseline vitamin D status, thereby diluting any treatment effect confined to individuals with deficient vitamin D status. This review does not inform whether supplementation reduces depression, only that deficiency is associated with increased depression risk in longitudinal studies.

Four limitations require acknowledgement. First, residual confounding is probable: physical activity, sleep quality, inflammatory burden, and socioeconomic position covary with both 25(OH)D and depression and were incompletely or inconsistently measured. Second, assay heterogeneity (RIA, LC-MS/MS, CLIA, enzyme-linked immunosorbent assay (ELISA)) introduces inter-study measurement variance; RIA systematically overestimates 25(OH)D by 10%-15% relative to LC-MS/MS [31], which could misclassify borderline-deficient participants and attenuate apparent effects. Third, Egger’s test produced a p-value of 0.065; while non-significant, this signal merits acknowledgment. Formal assessment of small-study bias requires at least 20 studies for adequate power, and publication bias in the vitamin D-depression literature has been previously documented [32]. Fourth, the geographic evidence base, concentrated in North America, Europe, and East Asia, substantially under-represents sub-Saharan Africa, South Asia, and Latin America, regions with a high prevalence of vitamin D deficiency and a high depression burden but sparse longitudinal cohort data.

## Conclusions

The current body of longitudinal evidence consistently supports an association between serum 25(OH)D deficiency and increased odds of depression, with directionally homogeneous findings across diverse geographic regions, study populations, deficiency thresholds, and depression assessment methods. The complete absence of between-study heterogeneity, consistent direction across all subgroups, and robustness in sensitivity analysis strengthen the inference that this association is not an artefact of study design. However, the central clinical question, whether correcting confirmed serum 25(OH)D deficiency reduces incident or recurrent depression, remains unanswered. Randomised trials that pre-screen for deficiency, maintain double-blinding, and use incident clinical depression as the primary endpoint are required.

## Appendices

Appendix A

*Supplementary Analysis: R Code for Forest Plot and Meta-Analysis' Heading* library(metafor) # Study-level data dat <- data.frame( study = c( "Pan et al. [15]", "Hoogendijk et al. [16]", "Bertone-Johnson et al. [17]", "Armstrong et al. [18]", "Milaneschi et al. [19]", "May et al. [20]", "Cassidy-Bushrow et al. [21]", "Lee et al. [22]", "Schottker et al. [23]", "Shaffer et al. [24]", "Lapid et al. [25]", "Polak et al. [26]", "Zhao et al. [27]", "Jorde et al. [28]", "Hoang et al. [29]" ), OR = c( 1.53, 1.85, 1.75, 1.63, 2.06, 1.22, 1.28, 1.44, 1.41, 1.31, 1.38, 1.46, 1.57, 1.94, 1.42 ), lower = c( 1.19, 1.29, 1.12, 1.28, 1.30, 0.94, 0.97, 1.11, 1.19, 1.09, 1.02, 1.08, 1.21, 1.14, 1.07 ), upper = c( 1.97, 2.64, 2.73, 2.08, 3.26, 1.59, 1.69, 1.86, 1.68, 1.57, 1.86, 1.98, 2.03, 3.31, 1.91 ), n = c( 3000, 1282, 2823, 5401, 956, 3916, 2016, 4523, 9940, 7358, 1304, 1817, 3262, 441, 1892 ) ) # Calculate log odds ratios and standard errors dat$yi <- log(dat$OR) dat$sei <- ( log(dat$upper) - log(dat$lower)) / (2 * 1.96) # Random-effects meta-analysis using REML res <- rma( yi = yi, sei = sei, data = dat, method = "REML") # Model summary print(res) # Pooled effect cat( "\nPooled OR:", round(exp(as.numeric(res$b)), 3), "\n95% CI: [", round(exp(res$ci.lb), 3), ", ", round(exp(res$ci.ub), 3), "]\n" ) # Heterogeneity statistics cat( "\nHeterogeneity:\n", "I² =", round(res$I2, 1), "%\n", "Tau² =", round(res$tau2, 4), "\n", "Q-test p-value =", round(res$QEp, 4), "\n" ) # Forest plot par(mar = c(5, 6, 2, 2)) # Forest plot forest( res, slab = dat$study, refline = 0, xlab = "Odds Ratio (95% CI)", alim = log(c(0.5, 4)), at = log(c(0.5, 1, 1.5, 2, 3, 4)), atransf = exp, ilab = dat$n, ilab.xpos = -2.5, header = c("Study", "Odds Ratio [95% CI]"), cex = 0.8, psize = 1, col = "navy" ) # Add sample size header text(-2.5, 17, "N", font = 2) # Egger's regression test egger_test <- regtest( res, model = "rma" ) print(egger_test)
 
